# AutoRoot: open-source software employing a novel image analysis approach to support fully-automated plant phenotyping

**DOI:** 10.1186/s13007-017-0161-y

**Published:** 2017-03-08

**Authors:** Michael P. Pound, Susan Fozard, Mercedes Torres Torres, Brian G. Forde, Andrew P. French

**Affiliations:** 1 School of Computer Science, University of Nottingham, Nottingham, NG8 1BB UK; 2 Lancaster Environment Centre, Lancaster University, Lancaster, LA1 4YQ UK; 3 School of Biosciences, University of Nottingham, Sutton Bonington, LE12 5RD UK

**Keywords:** Image analysis, Phenotyping, Traits, Software, Automated analysis

## Abstract

**Background:**

Computer-based phenotyping of plants has risen in importance in recent years. Whilst much software has been written to aid phenotyping using image analysis, to date the vast majority has been only semi-automatic. However, such interaction is not desirable in high throughput approaches. Here, we present a system designed to analyse plant images in a completely automated manner, allowing genuine high throughput measurement of root traits. To do this we introduce a new set of *proxy* traits.

**Results:**

We test the system on a new, automated image capture system, the Microphenotron, which is able to image many 1000s of roots/h. A simple experiment is presented, treating the plants with differing chemical conditions to produce different phenotypes. The automated imaging setup and the new software tool was used to measure proxy traits in each well. A correlation matrix was calculated across automated and manual measures, as a validation. Some particular proxy measures are very highly correlated with the manual measures (e.g. proxy length to manual length, r^2^ > 0.9). This suggests that while the automated measures are not directly equivalent to classic manual measures, they can be used to indicate phenotypic differences (hence the term, *proxy*). In addition, the raw discriminative power of the new proxy traits was examined. Principal component analysis was calculated across all proxy measures over two phenotypically-different groups of plants. Many of the proxy traits can be used to separate the data in the two conditions.

**Conclusion:**

The new proxy traits proposed tend to correlate well with equivalent manual measures, where these exist. Additionally, the new measures display strong discriminative power. It is suggested that for particular phenotypic differences, different traits will be relevant, and not all will have meaningful manual equivalent measures. However, approaches such as PCA can be used to interrogate the resulting data to identify differences between datasets. Select images can then be carefully manually inspected if the nature of the precise differences is required. We suggest such flexible measurement approaches are necessary for fully automated, high throughput systems such as the Microphenotron.

**Electronic supplementary material:**

The online version of this article (doi:10.1186/s13007-017-0161-y) contains supplementary material, which is available to authorized users.

## Background

Phenotyping is the process of measuring features or traits of a plant’s appearance. This appearance is affected by the plant’s growth characteristics (as determined by its genome) and the effect of the environment (such as stress factors or nutrient availability); quantifying the phenome is therefore our gateway into understanding these hidden factors. In recent years, the field of software-assisted phenotyping for plants has advanced tremendously [1–3]. The need to measure more traits from plant images, using larger and more varied image datasets has driven the need to develop more resilient computer vision algorithms to assist with this process [4–6]. Tasks such as measuring architectural traits from images are extremely labour intensive, yet computationally challenging to completely automate. Approaches to date largely *semi*-automate the process (e.g. [7, 8]). This leverages the expert-biologist’s knowledge of the domain to guide a computer algorithm, which can take care of the low-level processing, making the biologist’s job easier—but not removing user involvement entirely.

The problems of shoot and root phenotyping present distinct challenges, as such many tools are developed with a focus on roots specifically. For truly automated phenotyping, bottom-up approaches are the most common. GiaRoots [9], EzRhizo [10], WinRhizo and RootReader2D are notable examples. These often apply a low level binary classification operation such as local or global thresholding, to determine which pixels correspond to root material, and which to background. The benefit of assigning labels to pixels in this way is that broad quantitative measures of root systems can be calculated quickly. Measurements such as inferred rootmass, width, tortuosity etc., are easily computed on binary images. However, it is often challenging to perform this thresholding in the presence of noisy images, and large amounts of processing can be required to clean up the signal, for example by removing anomalously-detected foreground pixels. In those cases where the removal process is not perfect, the resultant trait values become flawed. It is commonly held that this low-level error can be overcome by increasing the size of the dataset, something that can be done trivially in automated systems. This approach undoubtedly has merit, but the extent to which this is true in practice will often be a function of the input images, and of the traits being measured; care must be taken.

More recently, methods that adopt a machine learning approach to quantifying the images (e.g. [11–13]), have grown in popularity. Learning algorithms are able to effectively adapt parameters based on a training image set. However, they require an initial training phase on a new image set before they can be used. To generate the training data, an expert must label the data by hand to provide suitable ground truth data for learning.

Processing power is increasingly in demand for these approaches, as the image analysis methods become more and more complex. This is especially true with recent deep learning approaches [14, 15], which can require substantial GPU-based hardware systems to train the complex networks in a feasible time. Other, more traditional image analysis software can still make heavy demands on processing power. Online frameworks are available to help take some of the processing power requirements of these algorithms away from the user, and simplify the processing pipeline (e.g. the iPlant Collaborative Project [16]). Still, despite helping with raw processing power and providing a consistent user interface—both important requirements for successful real-world use of software—the underlying algorithms still require research and development to become fully automatic.

To adopt a fully automated phenotyping approach, any software must fulfil particular criteria. Once running it should not make any demands on the user; all images in the set must be processed in one go, as a batch. Previous software has taken this approach (e.g. [17]), requiring the user to provide some details to the software initially, but after that period, batch processing proceeds through an entire set of images. However, it is still beneficial to perform manual visual checking of the final results, to confirm whether the images have been successfully processed. At some point, though, this approach becomes infeasible. With the introduction of more capable robotics enabling very high throughput image capture, it becomes challenging to verify that all the results are satisfactory. We need to ask the software to either place a confidence in the measured results, or provide results which are inherently probabilistic. To say a root is 32.6 mm long requires much certainty on behalf of the software and developers, and the degree of this certainty is often not addressed in automated software. To say root A is longer than root B may be just as valuable, yet requires a looser set of processing requirements.

The software itself should run sufficiently fast so as to keep up with throughput of the image capture, or at least be able to batch process results offline and in time for the arrival of the next batch. With typical phenotyping studies requiring 1000s of images (e.g. [18]) the issue of processing speed is becoming increasingly important. This is the motivation behind the approach presented here. We propose a system designed explicitly to work with images generated in a high throughput manner using a robotic capture system. The software does not require pre-training per image set, as image capture settings are able to be kept consistent as part of the imaging setup. Processing requirements are sufficiently small that an image can be processed in a few seconds on a standard PC (i.e. faster than the rate of image capture). User interaction is not required during processing, and results are automatically generated.

The nature of the results is such that the necessity to manually validate the results is minimized; we propose measures in the next section which implicitly handle the inherent uncertainty in most image analysis approaches. To achieve automated phenotyping with measurable reliability, a new set of *proxy* measurement traits are proposed, described here. Traditional manual phenotyping involves measuring visible features of a plant using tools such as a ruler or protractor. Despite appearances, even these measurements are not *certain*; and at some degree of accuracy are always incorrect. Implicit in such measurements is an error: a user can only read a measurement to a certain level of accuracy, for example, or may miscount the number of leaves or lateral roots etc. Despite the error, we often consider such measures as a ‘ground truth’ or gold standard; that is, they represent a direct measure of the trait which we often consider to be error-free. Statistical methods applied to measurement data will reveal variations in measurement accuracy, but are considered post-measurement, rather than during the measurement process itself. The same is true of automated image analysis tools—thresholding applies a hard *root* or *no root* label to each pixel, which is sensitive to noise and lighting [19], and there is often little indication of the relative error on a particular image.

Our approach, built here into the new AutoRoot software, differs from traditional root analysis tools by first making no firm judgement as to the location of root pixels—i.e. a thresholding approach is not used. A shortest path-based approach instead measures the confidence that a given pixel is part of the root system, and to which plant it belongs. It is these confidences that drive our phenotypic measurements. In essence, each trait is calculated based on where the root material is likely to be. We will demonstrate that the new traits we propose to measure correlate well with typical manual measures, and can be used as further input in analysis approaches (such as clustering or more advanced machine learning based techniques) without any further interpretation necessary. We also show the measures are able to separate two experimental growth conditions in a new high throughput imaging setup, in a completely automated manner. We believe that these proposed probabilistic *proxy* traits, have application in automated phenotyping where measurement of direct traditional traits is challenging, or not possible.

## Implementation

Images of roots and shoots of Arabidopsis seedlings were acquired using an automated image capture system called the Microphenotron [20]. Briefly, the seedlings were grown in agar-filled ‘phytostrips’, which are transparent plastic growth devices that are comprised of a strip of eight flat-sided growth vessels. Growth conditions and nutrient media were as described by Forde et al. [21]. The internal distance from the front to the back of each growth vessel is 2 mm, obliging the root system to develop in an essentially 2-D conformation to facilitate imaging and image analysis. Images of the phytostrips are captured robotically, such that each image contains eight wells of seedlings. The precision of the robotic manipulation, combined with the physical boundaries of the growth vessel, places definite limits on where the plant material can be found in the image. In our system we use this consistency to limit our search for root material to these wells, however the methods we propose here would work equally well on larger images. Hereafter, where we discuss image analysis approaches, we are referring to the contents of a single well, and this process is repeated for the number of wells in the input image.

Images are prepared by first converting to greyscale, before contrast is increased by performing a contrast stretching operation. This maps a range of intensity values representing the background and foreground pixels, into the full 8-bit grayscale range of 0–255 intensity levels. In practice, this has the effect of increasing the grey-level distance between the foreground and background, improving the confidence measures that are calculated next. The Microphenotron has very consistent lighting environment, and as such these values are easily determined once before being applied to all images. It is not necessary to perform an adaptive method, such as histogram normalisation. We tested a histogram normalisation approach which redistributes grey levels in the image such that a small percentage of pixels are saturated at either the minimum zero value, or the maximum greyscale value. We found, however, that for dense root systems, histogram normalisation would produce insufficient contrast, spreading the intensity levels of root pixels over the full range, rather than significantly brightening them.

Rather than performing a binary threshold, which assigns a 100% confidence value to each pixel’s classification, we aim to calculate a ‘likelihood’ that any given pixel belongs to the root system. We begin by locating numerous candidate root locations within a horizontal strip at the top of each image well. The height of this strip is 5% of the well height. Seeds are planted above this position in all images we have encountered. This is done by finding local intensity maxima (pixels brighter than their neighbours) by scanning across each pixel row. These positions act as points at which we are confident there is root material, and thus points well connected to these will have a high likelihood of also being root.

These candidate points are used to initiate a Dijkstra’s shortest path search [22] through all pixels in the well. Dijkstra’s algorithm will find the shortest path between nodes in a graph. We consider the pixels inside the well regions as a fully-connected set of nodes. When run to completion, it is also possible to calculate the length of a path from any source nodes, to any point on the image. Our Dijkstra approach is similar to that found in our existing RootNav tool [7]; a graph is produced with a node at each pixel, and edges between neighbouring pixels. Unlike RootNav, where edges are weighted based on the output of an expectation–maximisation classification step, the weights in this system are based solely on the grayscale intensity of the pixels. In short, bright pixels will generally have low, favourable weights, and darker pixels will have higher, less favourable weights. Weights are calculated as:

1 $$w(p,q) = 1 - \alpha \cdot I(p) \cdot I(q),$$

where *p* and *q* are the two pixels between which the weights are calculated. *I*(*x*), is the normalised intensity of the image at pixel *x*, where 0 ≤ *I*(*x*) ≤ 1. The additional weight *α* is 1 if the pixels are in the same row or column, and $\sqrt 2$ if they are on a diagonal, to account for the increased distances between the pixel centres.

This results in Dijkstra’s algorithm finding shorter paths through bright regions of the image, and longer ones through dark regions; but note we require no arbitrary intensity boundary between foreground (root) and background (well). Simply, the algorithm penalises travel over non-root material.

Since our approach obtains a number of candidate source points representing the top of the root system, we adapted the Dijkstra implementation to consider termination at any one point in a set of points, rather than at a single point. The output of this step is a value, for each pixel, representing the distance required to travel from the source points at the top of the well, to each pixel’s position, taking account the weights as described previously. It is these distances that we treat as likelihoods in our probabilistic traits, under the assumption that shorter paths travel over brighter pixels (more likely root material). This approach has the added benefit of ignoring noisy bright pixels that are unconnected to the root structure—to reach these pixels the shortest path must travel over background, and thus the lengths of paths to noise are often significantly longer. Where noise appears close to the root system, this may not always hold; however we have observed this infrequently during our experiments.

**Fig. 1:**
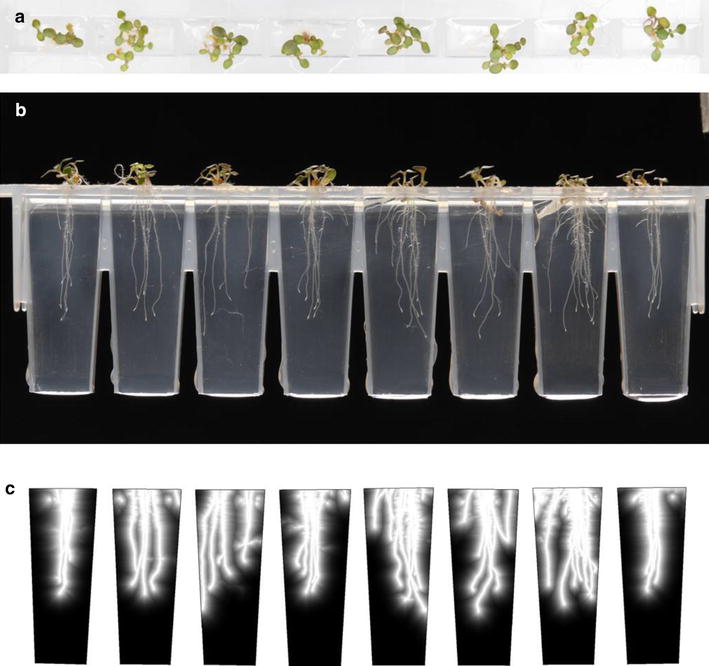
**a** Top-down image from the Microphenotron. **b** Side-facing image of the same plants in **a**. **c** *L*(*x*, *y*) visualised as a heat map for the set of eight wells in **b**. *Brighter areas* indicate regions the software considers more likely to be root material

In order to quantify phenotypic traits within each image or well, we define a likelihood function *L*, that for any pixel produces a likelihood that it belongs to the root system:

2 $$L(x,y) = 1 - dijkstra(x,y) /max(dijkstra(u,v),\quad \forall u,v)$$

where dijkstra(*x*, *y*) is the shortest distance to pixel *x*, *y*, from any start position (see Fig. 1). The value *L* is normalised using the maximum theoretical value of distance for an image of that size. In practice this can be calculated as the maximum distance returned by Dijkstra, averaged over a number of wells. Optionally, the function *L* can also be raised to a power, i.e. *L* ^*n*^ to decrease the distance at which the likelihood drops off from bright pixels. We have found this has minimal impact on results, but can be useful if plants are more established, and thus the maximum distance from root material is never large.

The function *L* indicates a likelihood of there being a root at a given location. Note, we are not thresholding the image at this point, rather we wish to assign a confidence level to represent how certain we are that this pixel belongs to a root. Any subsequent trait derived from this data can maintain the idea of this confidence. Therefore, we avoid the problematic situation of having to determine a priori the exact existence or non-existence or root at each location in the image, and can instead make an educated guess.

Once image pixels are assigned a confidence level indicating root material presence, and a distance to the determined anchor point, a number of interesting, but non-traditional traits can be measured from the image. They are measured in the image space (i.e. in pixels) but as we will see this is not important and conversion to real-world units is not necessary. The measures currently implemented in AutoRoot are presented in Table 1.

**Table 1:** Proxy traits proposed here

Proposed trait	Description	Nearest traditional equivalent
Centroid	The weighted centre of mass of the root system	Centre of mass of all root pixels
Mass	A normalised sum of all likelihoods generated by *L* in a given image or well	Sum of all root pixels
Width/depth (M)	Bounding box width and depth of the root system. Calculated as maximum point of mass on the *extremities* of the root system. We define this as: $\arg \max_{x} (L(x,y) \cdot x )$ and similarly for y and the other sides of the bounding box	Maximum width and depth reached by all root pixels
Width/depth (p95)	Alternative bounding box width and depth of the root system. Calculated to enclose 95% of the calculated root likelihood	Maximum width and depth reached by all root pixels, discounting a small number of root outliers
Depth (p99)	Alternative depth measurement, calculated as 99% of the root likelihood	Maximum depth reached by all root pixels, discounting less outliers than p95
Quadrant mass	The mass trait split horizontally into four regions for each well, giving a measure of root material within each quadrant (see Fig. 2a)	Root pixel count in four regions (at varying depths)
Orientation	Ten brackets of orientation representing the direction of the root system at each pixel. These range from 0°, horizontal, to 90°, vertical. (see Additional file 1: Figure S1)	Histogram of all root angles
Quadrant orientation	The orientation trait split horizontally into four regions for each well (as previously). Orientations are now grouped into four brackets per quadrant, rather than 10, giving 16 values for each well in total	Histogram of root angles at different depths
Leaf hue	The average hue for each leaf pixel in the top image, for each well	Average pixel hue i.e. leaf colour
Leaf area	Total pixel count for all non-white pixels in the top image, per well. Non-white is defined as having a saturation value above a low threshold of 20%	Pixel count of leaf area

Where appropriate, a nearest traditional measure is listed. *L* refers to the root likelihood function (Eq. 2). Note the final two traits are measured from a top-down camera view

These traits are calculated as a weighted function (e.g. a sum or average), where the contribution of each pixel to the final measurement is given by its likelihood. So rather than, for example, measuring the mass of the root system by summing over all thresholded foreground pixels, we sum the likelihoods of all pixels in a well. This means that all pixels are considered within each measured trait, but that those with high likelihood of being root material will make a significantly higher contribution. The orientation $\theta$ at each pixel is calculated using a Sobel convolution:

3 $$\theta = \tan^{ - 1} (Gy/Gx),$$

where Gx and Gy are the gradients in the *x* and *y* directions, calculated using convolution with a Sobel operator.

## Implementation and performance

AutoRoot runs in real time, typically taking a few seconds to process each well, and approximately 15 s to process each image on a standard, modern machine. The size of the images we used in this experiment were 5 MP, with each well approximately 290 px wide and 710 px tall. For images of this resolution, the software comfortably runs using <2 GB of RAM. The AutoRoot software has been developed in C#, and as such runs on any Windows installation. The full code is available under an open-source license.

## Results

In the following experiments we examine how useful the new metrics are as proxy measures for classically measured traits via a simple phenotyping experiment. We also consider the usefulness of using the new proxy measures directly as ways of discriminating different phenotypes. We have purposefully chosen clearly visible and distinct phenotypes in order to demonstrate both the process and descriptive power of the proxy traits. AutoRoot has been used successfully to recover more subtle phenotypic differences in an Arabidopsis root and shoot chemical screening experiment [20].

*Arabidopsis thaliana* seedlings were germinated and grown in phytostrips filled with nutrient agar (a 20-fold dilution of Gamborg’s B5 medium pH 5.7, 0.5 mM KNO_3_, 0.5% sucrose and solidified with 0.7% Phytagel). Treatments were applied using the approach previously described [23], where the phytostrips are placed in the wells of a microtitre plate containing 150 ul nutrient solution, with or without 40% (w/v) polyethylene glycol 8000 (PEG). Inclusion of the PEG in the microtitre plates is designed to impose a water-deficit stress on the growing seedlings, a treatment that is known to inhibit Arabidopsis root growth [23].

For the imaging, plants were automatically imaged in clear wells of growth media, using the Microphenotron system [20] (see Fig. 1).

### Automated measures as proxies for manual metrics

**Fig. 2:**
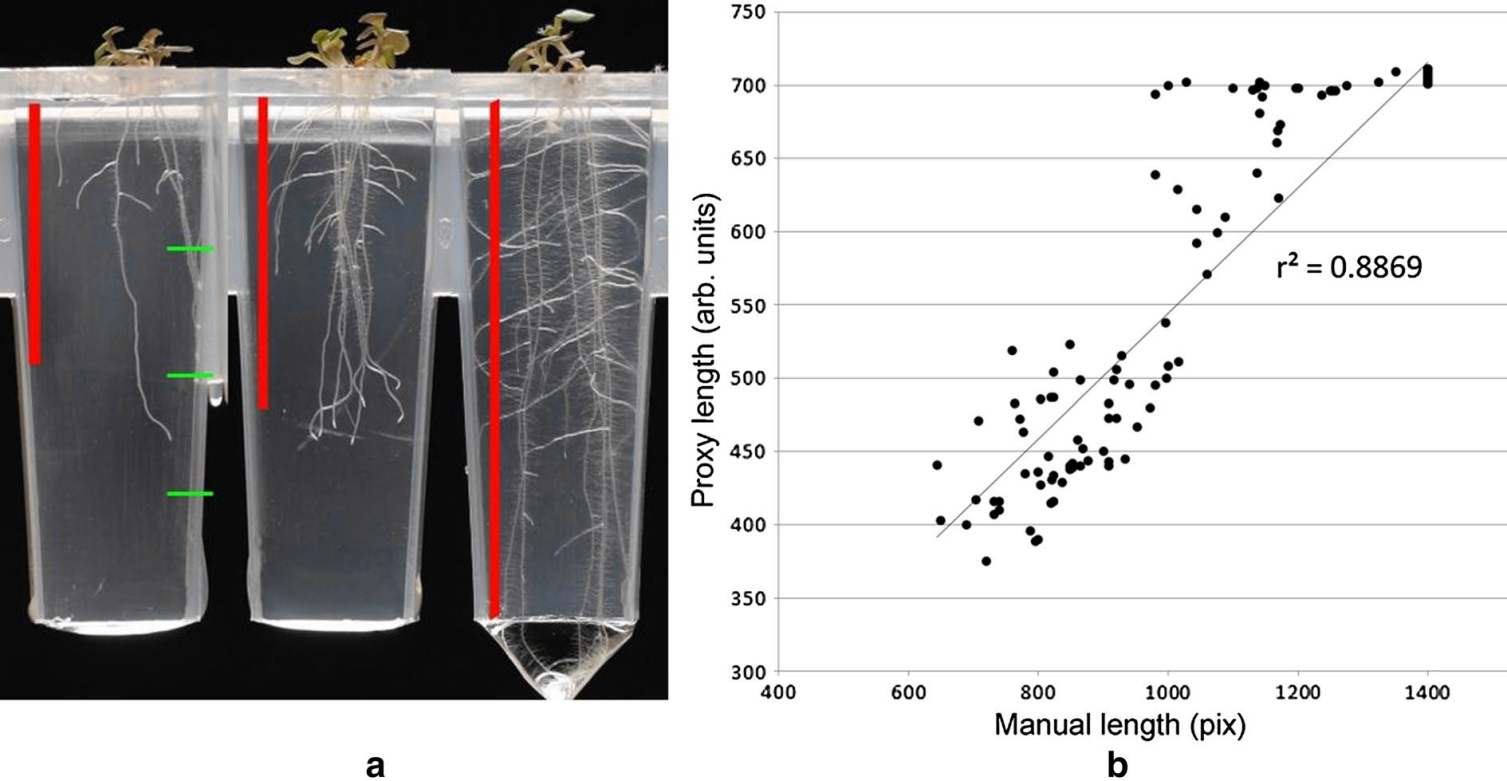
**a** Manual measures derived from three varying wells—*red bar* indicates manual root length estimations. *Green ticks* indicate approximate boundaries for quartile divisions—Q0 at the *top*, Q3 at the *bottom*. Comparison of manual and automatically derived measures for these wells can be seen in Table 1 and panel. Note the subjective judgment required for root length in the left hand well. **b** Manual length plotted against *proxy*-depth for a mixed population of plants in 184 wells. To note in the graph, some of the plants had reached the *bottom* of the wells, represented at the cluster at (1400, 700)

The measures that we propose can act as proxy measures for the more classical manual measures of the phenotype. To demonstrate this, plants in 23 images, each featuring eight wells, and grown in multiple conditions as described above (so expressing a variety of phenotypes) were both measured manually, and analysed with the new software. Manual measurements were carried out using the Fiji software (http://fiji.sc/) [24]. As wells contained multiple plants, a simple and fast approximation method was used to manually quantify root length, lateral count and estimate mass per well (inspired by the fast ‘shovelomics’ approach developed for in-field phenotyping [25]). To measure length, a representative root length was estimated for the well, using the straight line measuring rule in Fiji (see red bars in Fig. 2a for examples). Root density (sometimes termed mass, or abundance of root material) was estimated by categorising the well depending on the amount of root material present: 0 representing no growth at all, and 10 representing an abundance of root growth. The number of laterals in the well was categorised into bins (0–5, 6–10, 11–15, 16+). This is a coarse measure, but identifying individual laterals, and judging whether a particular section of root is lateral or not can be subjective, so no finer granularity was sought. Table 2 shows example measures (automatic and manual) from the wells in Fig. 2a.

**Table 2:** Well label corresponds to well from Fig. 2a

Well	Manual length	Manual density	Manual laterals	AutoRoot depth (M) (*proxy*-*length*)	AutoRoot mass (*proxy-mass*)	AutoRoot orientation 1 (*proxy-laterals*)
Left	644	1	0	441	54,313	76
Middle	852	5	10	442	75,919	297
Right	1400	9	15	709	153,493	1632

Manual measures are presented first, followed by automatically-derived proxy equivalents. Note proxy traits have arbitrary units

In order to examine the relationship between proxy parameters and manual measures, a correlation matrix was calculated between all proxy measures and manual measures for this dataset (see Table 3).

**Table 3:** Correlation matrix between a subset of the metrics, showing the highest-correlated proxy measures (in italics) with each manual measure

	Length	Density	Laterals
Centroid Y	*0*.*95*	0.71	0.79
Q3 mass	*0*.*94*	0.71	0.76
Depth (M)	*0*.*94*	0.70	0.79
Depth (p95)	*0*.*94*	0.70	0.79
Depth (p99)	*0*.*92*	0.69	0.78
Mass	0.91	*0*.*79*	*0*.*83*
Orientation: 2	0.86	*0*.*79*	*0*.*83*
Orientation: 3	0.83	*0*.*81*	*0*.*84*
Orientation: 4	0.80	*0*.*83*	*0*.*86*
Orientation: 5	0.72	*0*.*85*	*0*.*84*

For the manual measurement of length, the most correlated proxy measures are proxy-depth, centroid-Y and the vertical extent of the bounding boxes containing 95 and 99% of root material (Table 3). Q3 mass also provides a good correlation—this represents the amount of root material in the third horizontal quarter down the well. Intuitively, the amount of root material in the third and fourth quadrants will increase with the overall length of the root system, dependent on the particular length of the root system. Figure 2b shows proxy depth plotted against manually estimated length, showing a good correlation throughout the range of data.

The manual density measure correlates highly with both overall mass, and orientation measures which capture lateral root emergence, as these represent much of the root mass in a dense image. The manual lateral count again correlates highly with root mass at orientations 3–5 (which mainly capture near-horizontal to 45° lateral root material), and correlates well with mass (as much of the mass of the root system is given over to lateral roots).

Correlation is important as we are not measuring pure, classic traits here. Therefore if we can identify a proxy measure (or combination of measures) which correlate well with a classical trait, then these measures can be used to identify the phenotypes of interest. A strong positive correlation will show that as proxy measure A increases, this also indicates an increase in classical trait A, so phenotypes can be compared relatively. Hence the need to calibrate measures into real world units is not required.

Finally, note that the specific subset of proxy traits which correlate well with classical measures of interest will vary dependant on the exact nature of the phenotype, and so you may experience different correlations to those listed here. This is the motivation behind directly using the proxy measures as phenotypic discriminators, as investigated in the following section.

### Proxy measures as discriminators

**Fig. 3:**
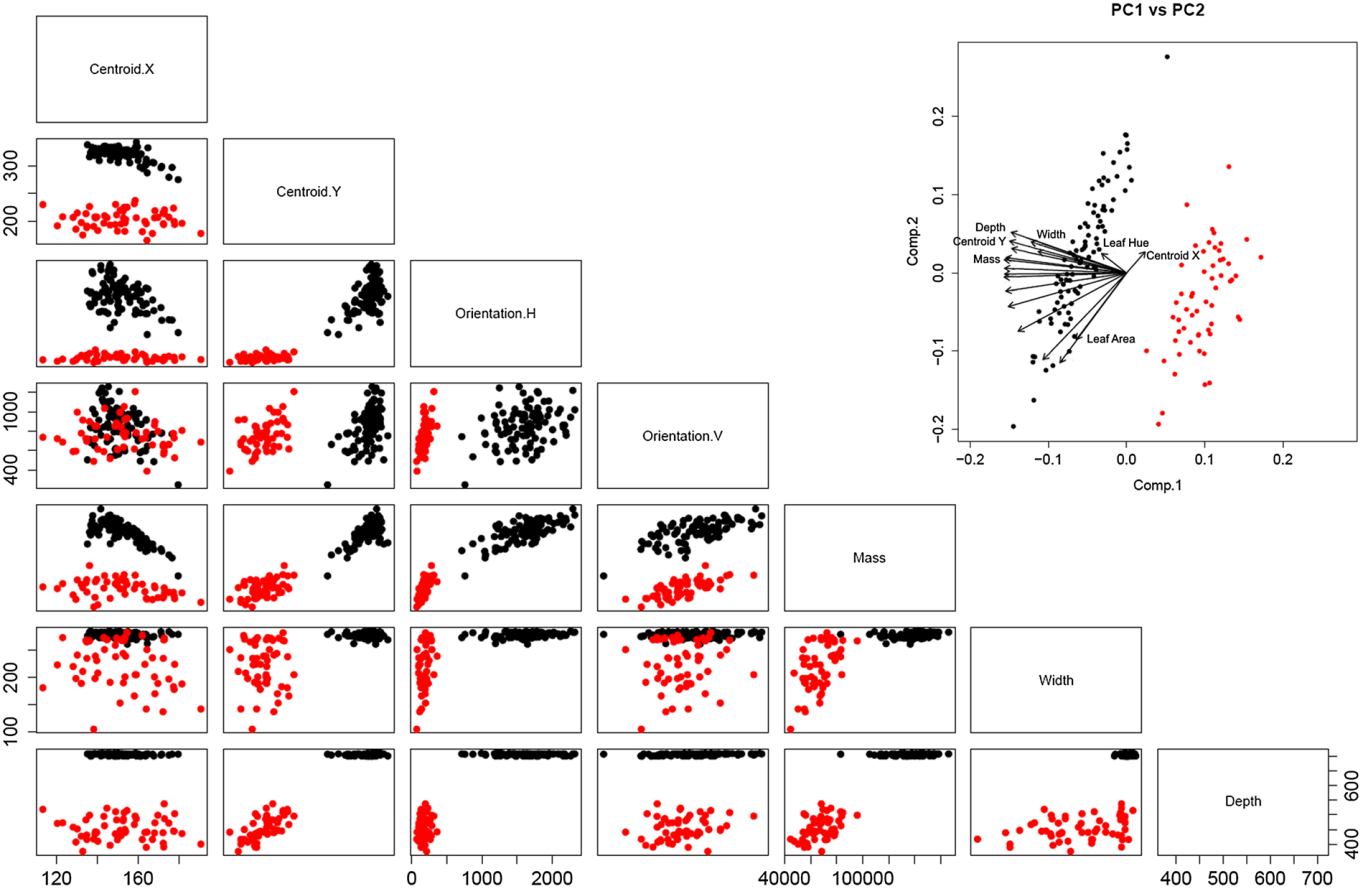
*Scatter plots* for pairs of proxy traits, for PEG (*red*) and control (*black*) datasets. *Inset* biplot of the PCA across components 1 and 2. Note how many of the proxy traits clearly separate the two experimental conditions. Therefore, they can be used to detect phenotypically different datasets directly (see Additional file 2: Figure S2 for example images)

As well as being correlated with traditional traits, the new proxy measures are useful discriminative measures in their own right. To demonstrate this, we analysed the raw data produced by the software from the same growth experiment described above. All well plates in all images were analysed, fully automatically without user interaction. Scatterplots of pairs of proxy measures (commonly referred to as a pairs plot) were produced (Fig. 3). This figure gives a helpful indication of how useful the measures are in identifying phenotypic differences in our example dataset. As can be seen in Fig. 3, the two growth conditions are strongly separable in many of the newly proposed measures. Based on this, traditional unsupervised clustering approaches such as k-means would work effectively in separating phenotypic classes.

Often, if individual traits do not provide enough separability, or some measures (such as variants on depth) are strongly correlated, or the dimensionality of the dataset is large, principal component analysis (PCA) can be used to perform dimensionality reduction. PCA provides a new set of variables that are created by linearly combining the original variables and maximising the variance of the dataset. The inset in Fig. 3 shows a biplot of the results of PCA on this dataset, for principal components 1 and 2 (the components with the largest proportion of the variance of the data). Together, these components represent over 78% of the variance in this dataset, and can be shown to separate easily the control and treated plants.

## Conclusion

In this paper we present an alternative to traditional manual image measures, which we term *proxy traits*. They can be calculated over complex images where segmenting all parts of a root system is not possible, or not reliable. These proxy traits are well suited to fully automated analysis settings, especially as part of automated robotic-based systems. Computational performance exceeds image capture, so does not produce an additional bottleneck.

As shown in the results, the proposed proxy measures are able to correlate well with existing metrics commonly derived from image data. This suggests these fast-to-calculate and automatically-derived measures are suitable for replacing some of these traditional metrics, and can be used to identify relative differences in phenotypic conditions.

In addition, analysis of the proxy measures via statistical techniques such as PCA and visualisation using scatter plots reveals that it is possible to directly discriminate between different plants exhibiting different phenotypic traits. As can be seen from Fig. 3, many of the proxy traits separate across the two datasets, showing that they can be used to identify phenotypic differences between the plants. Of course, for more subtle phenotypic differences, the separation may not be as dramatic, and only some traits (or combination of traits) may separate conditions. If this is the case, usual statistical treatments can be applied to the proxy measures to identify subtle phenotypic differences, just as with traditional measures.

The new measures are not a suitable alternative to manual or semi-automatic approaches, where fine-grained phenotypic analysis is required. However, we have shown that they can be used as an alternative to many traditional high-throughput measures, and that they are able to separate phenotypically-different datasets. In many cases (e.g. [7]) fine-grained capture of a root system architecture is used as an intermediate step to producing phenotypic traits, in which case this intermediate step could be skipped, significantly speeding up image analysis. Finally, there is no reason to believe similar traits could not be used in phenotyping other systems, such as plant shoots, where similar proxies could be used.

## Additional files

**Additional file 1: Figure S1.** Histogram of the relative frequencies of orientations in control and treated.

**Additional file 2: Figure S2.** Example control and treatment images.
