# Supplementary material for: AutoRoot: open-source software employing a novel image analysis approach to support fully-automated plant phenotyping
Source: Plant Methods. 2017 Mar 8;13:12. doi: 10.1186/s13007-017-0161-y (PMC5341458; doi:10.1186/s13007-017-0161-y)
Supplement: Supplementary file 1 — Additional file 1: Figure S1. Histogram of the relative frequencies of orientations in control and treated. [file 13007_2017_161_MOESM1_ESM.docx]

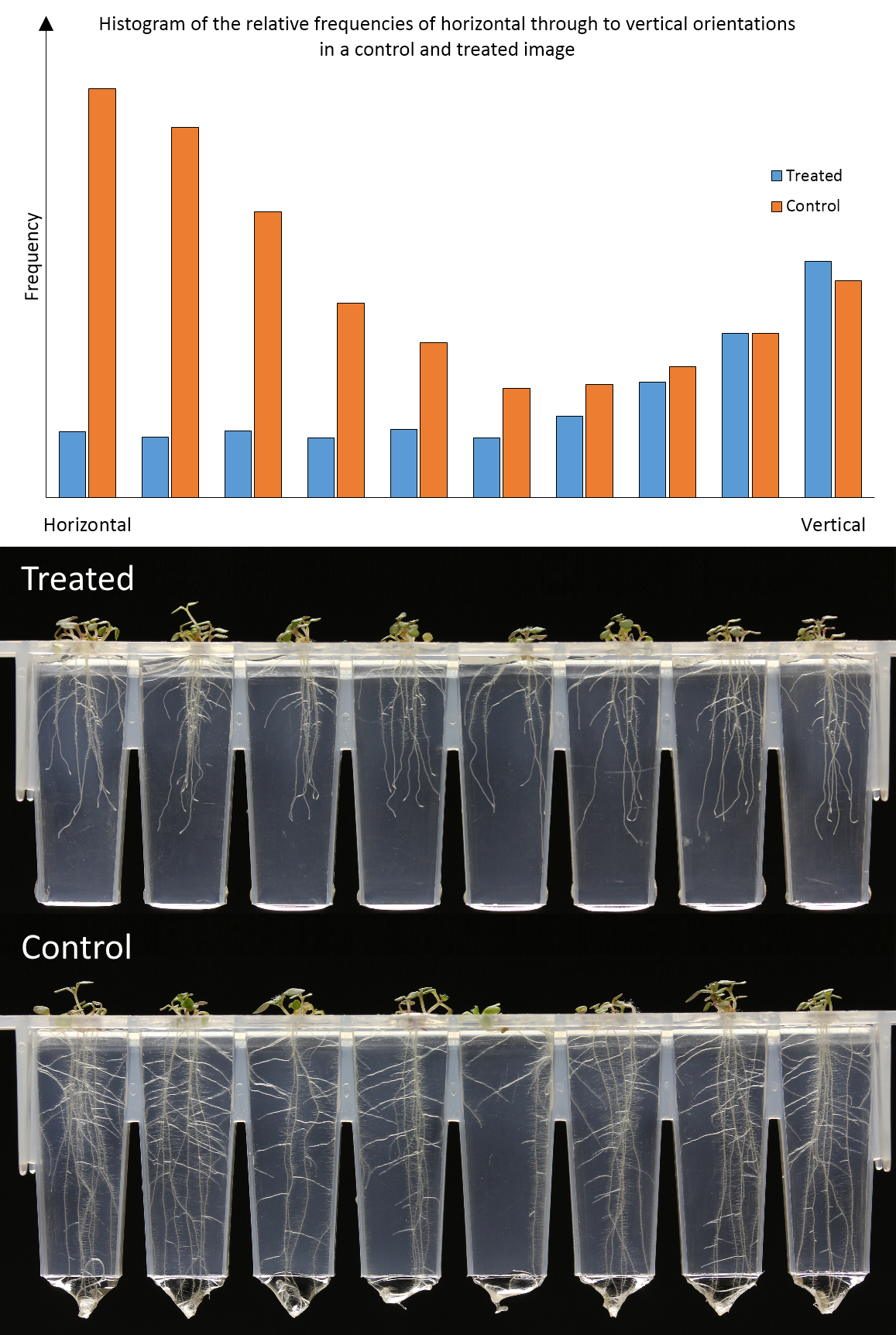


Additional file 1: Figure S1. A histogram visualising the distribution of horizontal and vertical root material averaged over 8 wells. The control plants are larger and more established, containing a great deal more horizontal root material.
