# Supplementary figures and images for: AutoRoot: open-source software employing a novel image analysis approach to support fully-automated plant phenotyping
Source: Plant Methods. 2017 Mar 8;13:12. doi: 10.1186/s13007-017-0161-y (PMC5341458; doi:10.1186/s13007-017-0161-y)

| **Control** | **Treatment** |
| --- | --- |
| 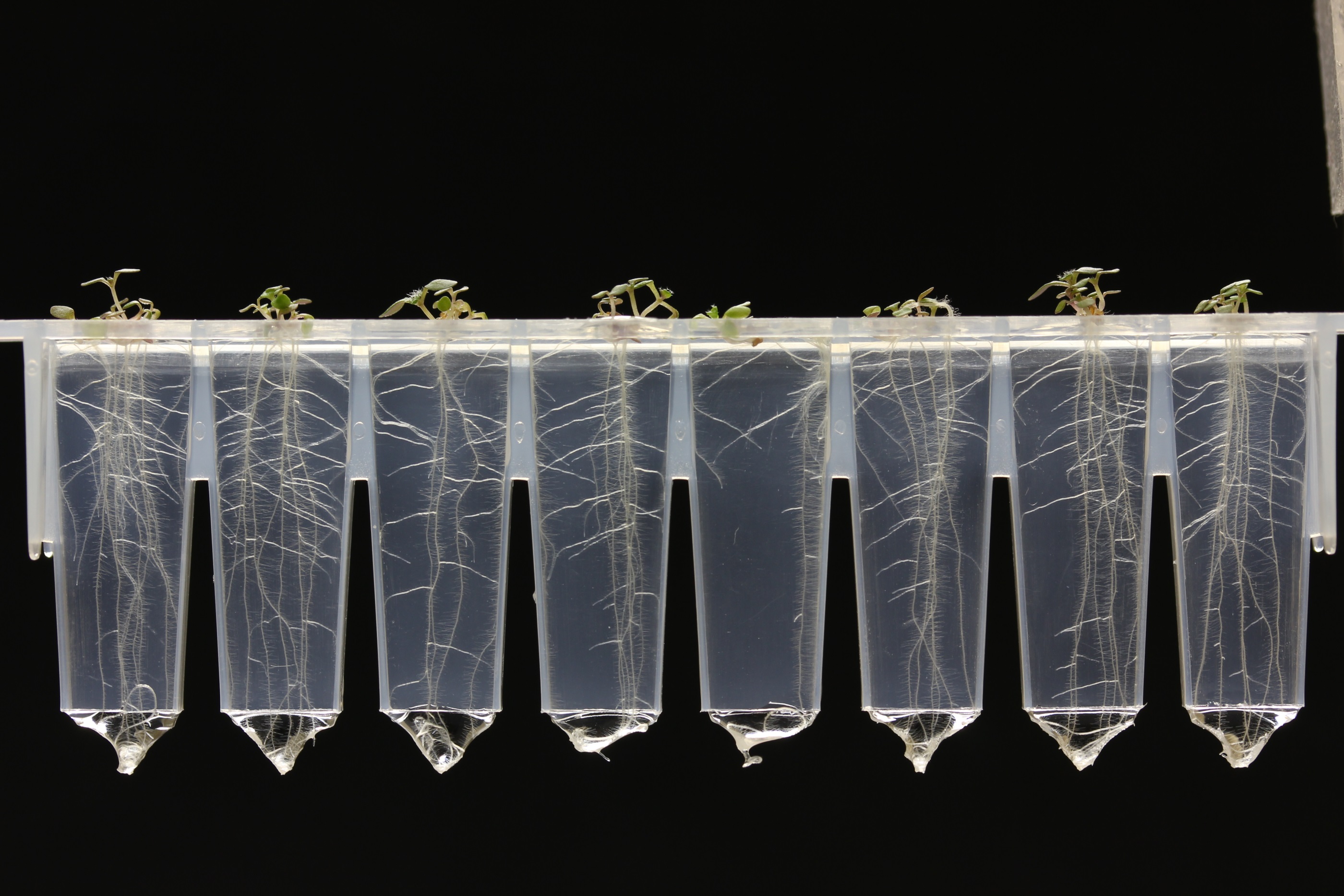 | 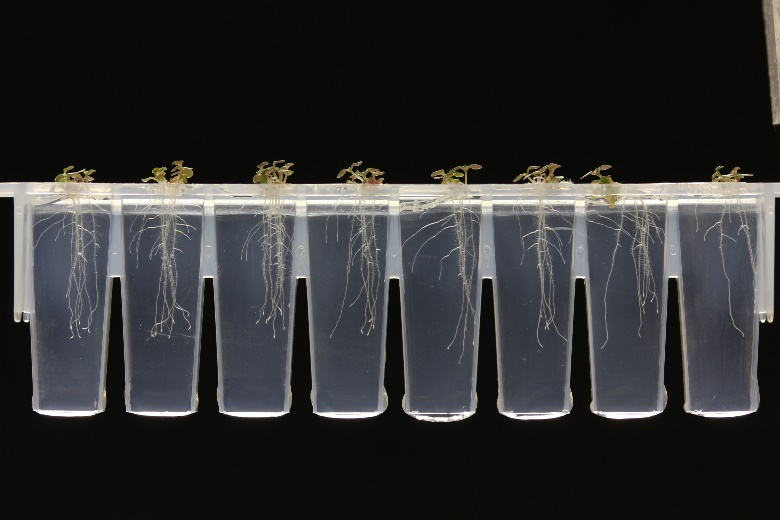 |
| 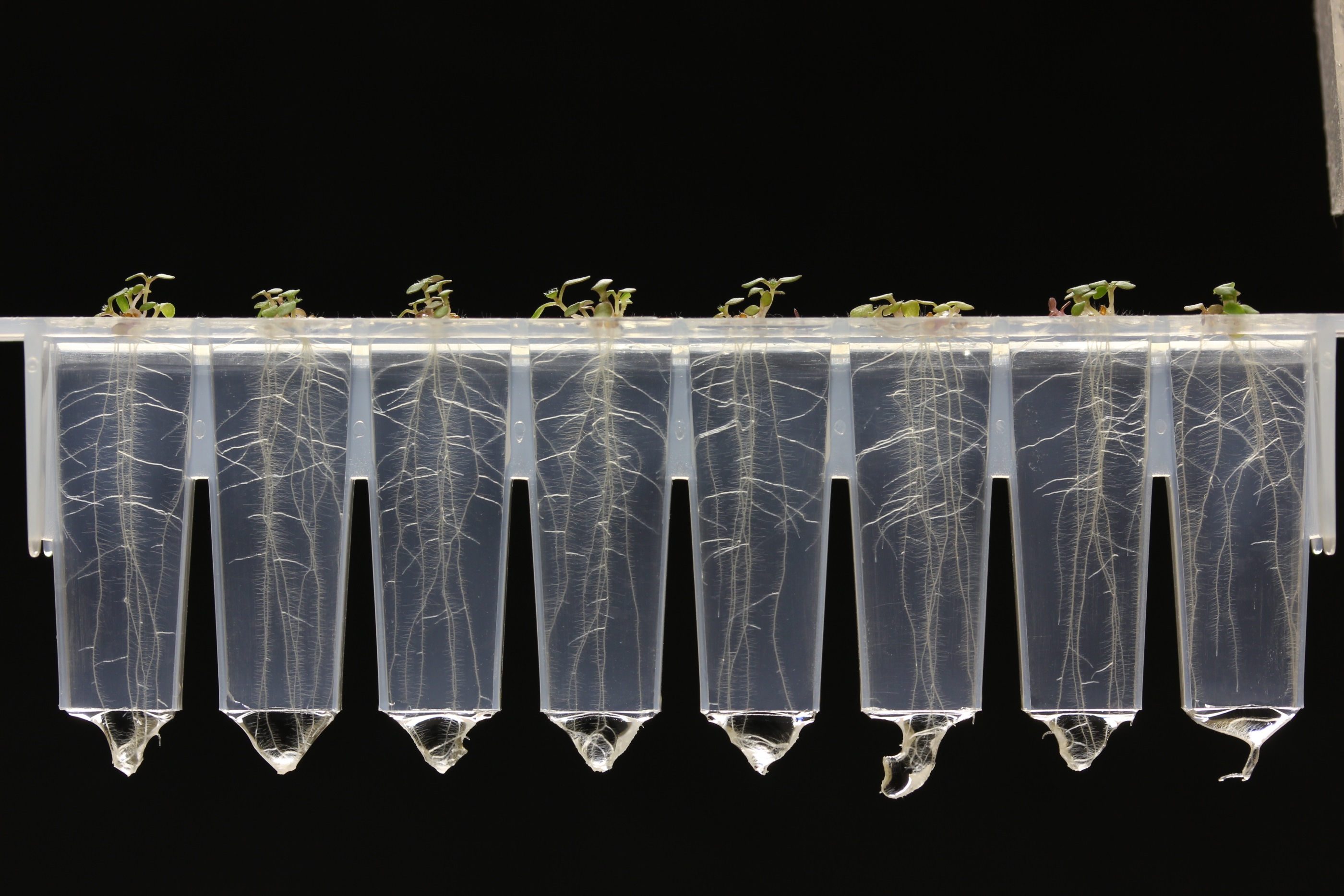 | 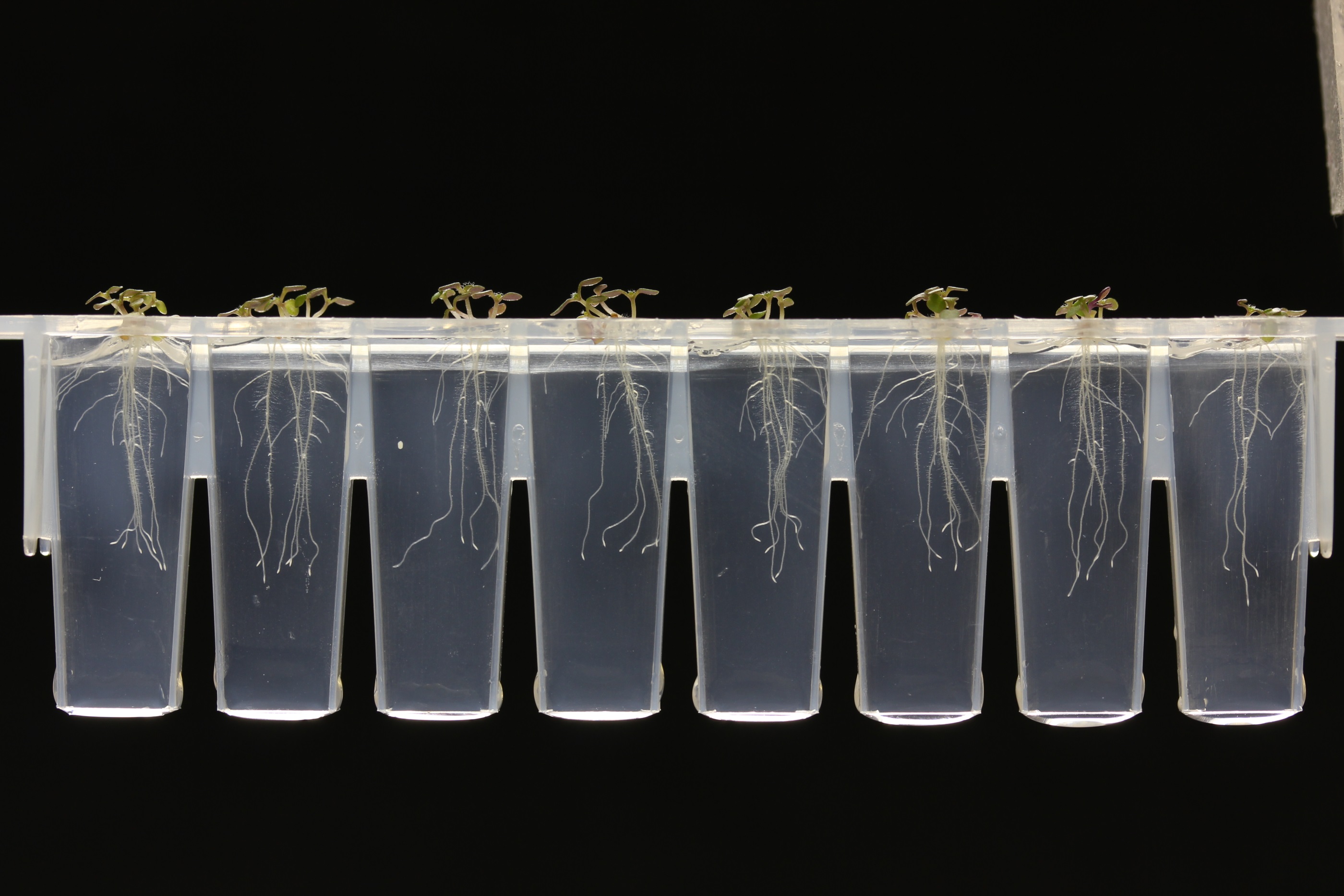 |
| 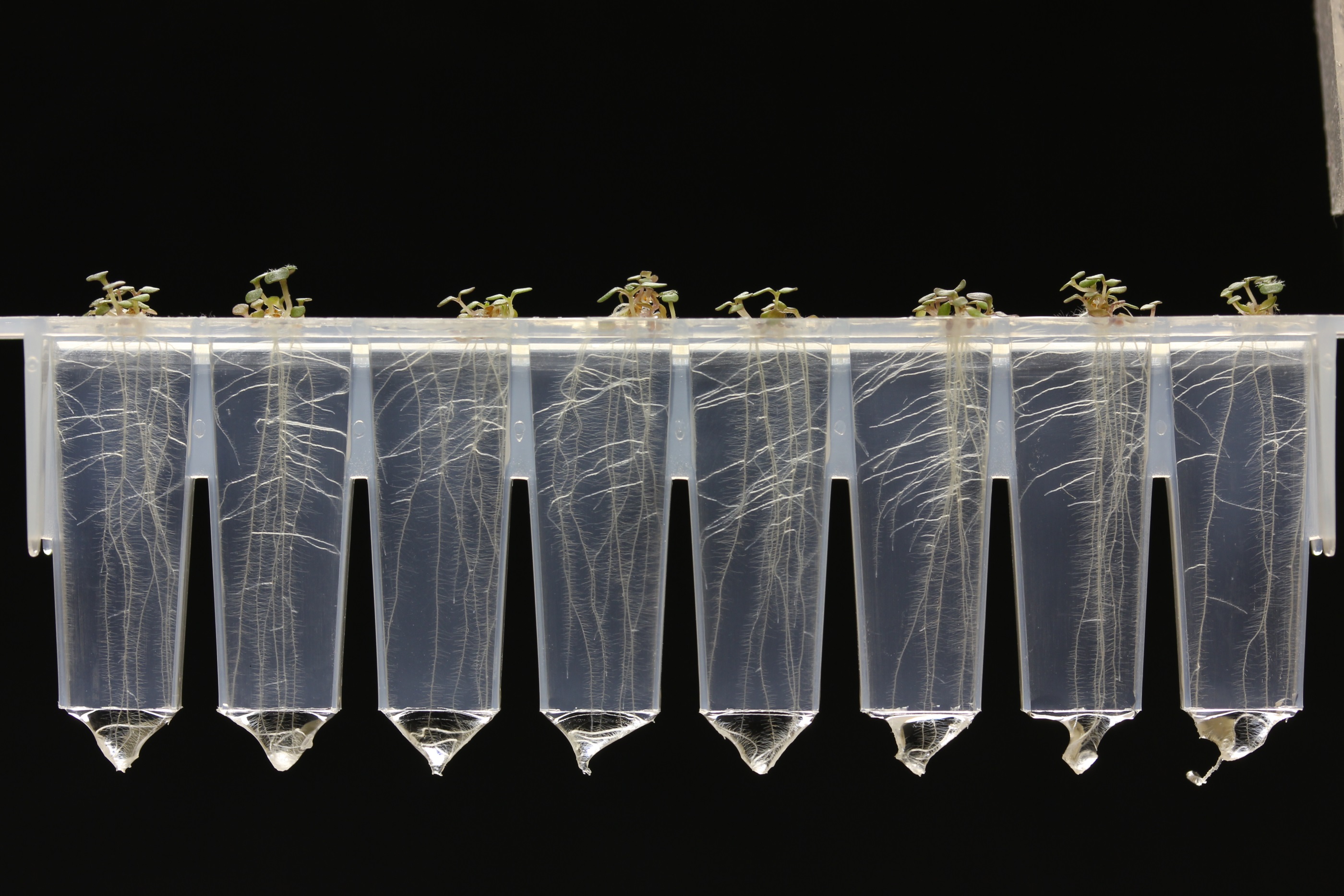 | 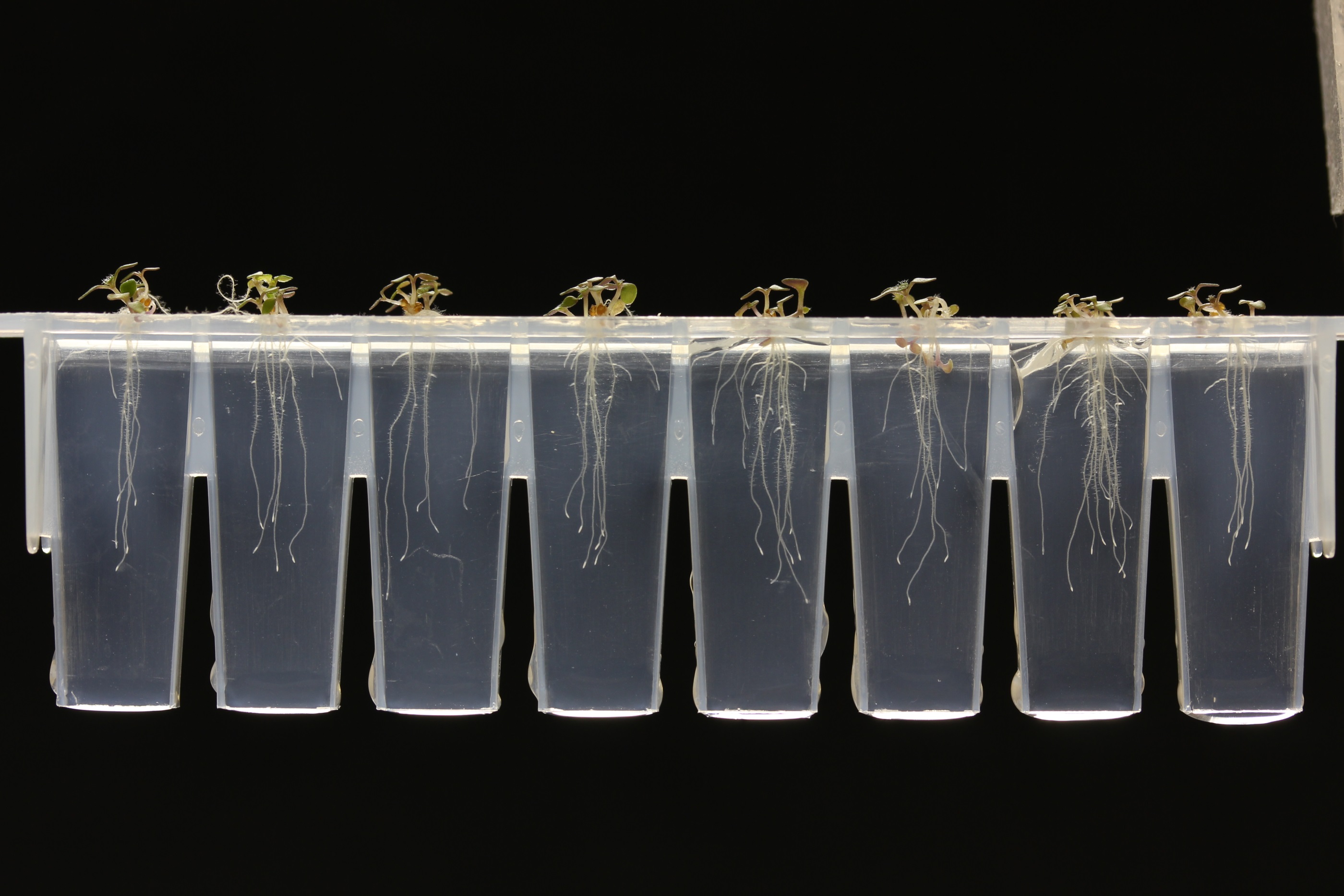 |
| 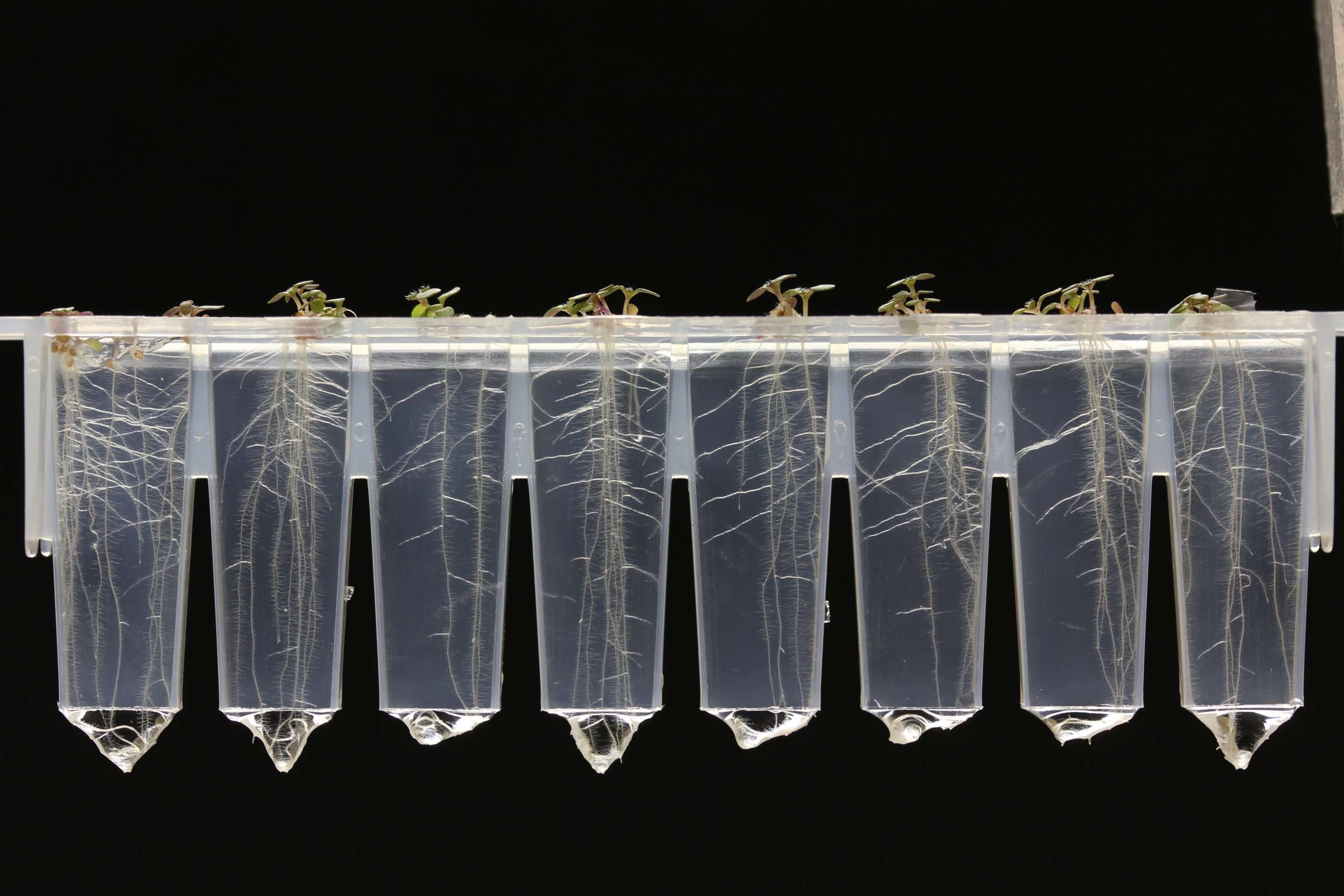 | 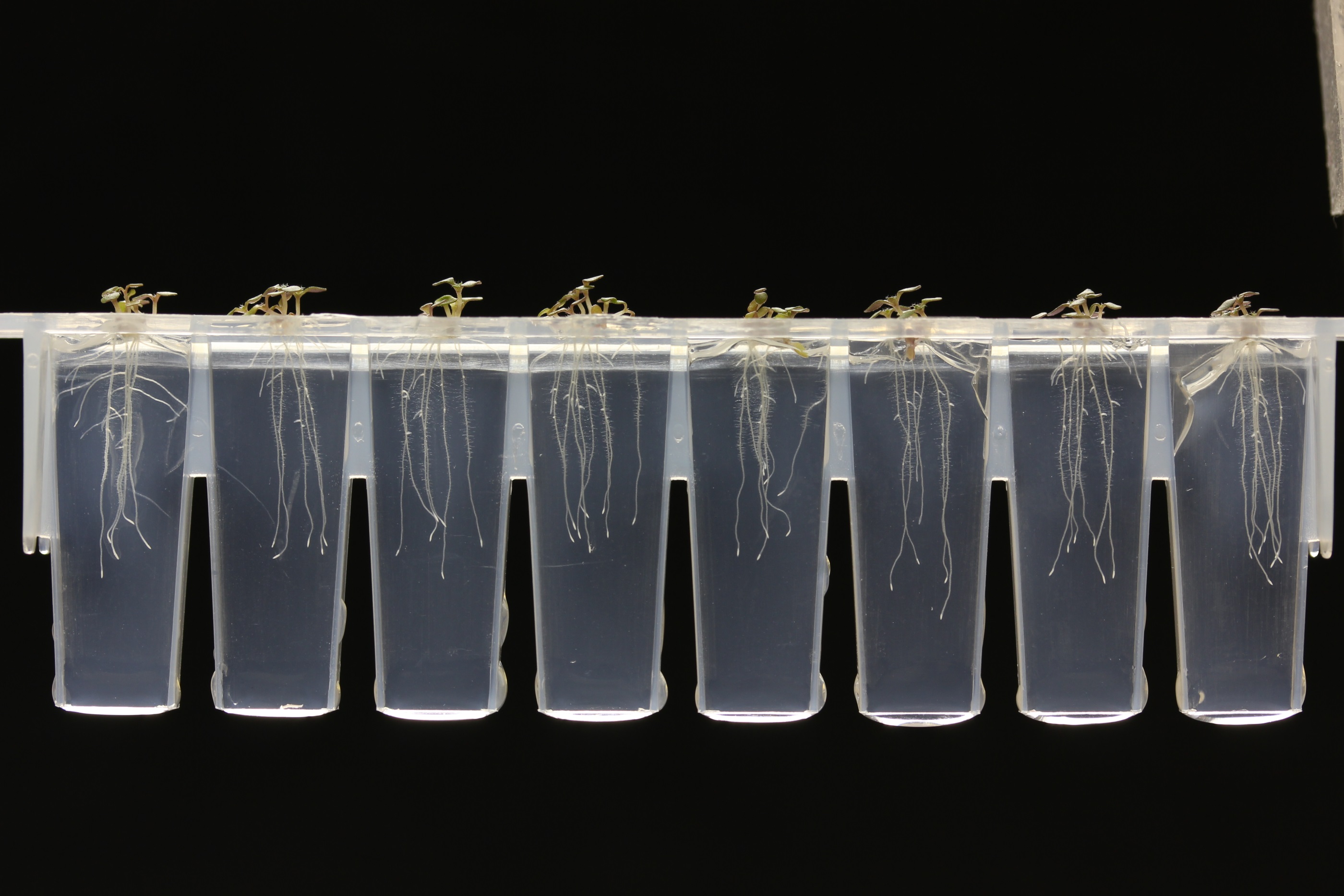 |

Supplement: Supplementary file 2 — Additional file 2: Figure S2. Example control and treatment images. [file 13007_2017_161_MOESM2_ESM.docx]
